# The Secondary Manifestation of a Marginal Zone Lymphoma in the Larynx: Lessons From a Misdiagnosis

**DOI:** 10.7759/cureus.13410

**Published:** 2021-02-18

**Authors:** Rakan Saadoun, Eva-Maria Risse, Theresa Obermueller, Ranim Bittar, Christoph Aderhold

**Affiliations:** 1 Department of Otorhinolaryngology, Head and Neck Surgery, University Medical Centre Mannheim, Mannheim, DEU; 2 Faculty of Medicine Mannheim, Ruprecht Karls University Heidelberg, Mannheim, DEU; 3 Department of Hand, Plastic and Reconstructive Surgery, Berufsgenossenschaftliche (BG) Trauma Center Ludwigshafen, Ludwigshafen, DEU; 4 Faculty of Medicine Heidelberg, Ruprecht Karls University Heidelberg, Heidelberg, DEU; 5 Department of Otorhinolaryngology, Head and Neck Surgery, Charité-Universitätsmedizin Berlin, Freie Universität Berlin, Humboldt-Universität zu Berlin, and Berlin Institutes of Health, Berlin, DEU; 6 Department of Cardiology, Heart Center of Riverside, Riverside, USA

**Keywords:** primary endolaryngeal lymphoma, secondary endolaryngeal lymphoma

## Abstract

The secondary manifestation of a marginal zone lymphoma (MZL), which is a less common type of B-cell non-Hodgkin’s Lymphoma (NHL), in the larynx is a rarity. We report a case of the secondary involvement of the larynx following MZL in a 72-year-old woman who presented with the sensation of a foreign body in the throat and history of MZL. A fiberoptic laryngoscopy confirmed the presence of a mass in the supraglottic area. The initial clinical evaluation indicated that the mass was benign. Hence, it was removed surgically. However, the histopathological analysis confirmed the diagnosis of MZL. After a systemic evaluation, the patient was classified as stage IV A according to the Ann Arbor staging system for lymphomas. Treatment was initiated with Ibrutinib 520mg/d and Rituximab 375 mg/m^2 ^every 28 days. When it comes to tumors of the head and neck, including NHL, the larynx should also be considered as a site for a possible secondary as well as primary involvement.

## Introduction

NHL of the head and neck can be divided into primary and secondary in terms of the original location of the lymphoma. The manifestation of NHL can be further divided into nodal, lymphatic extranodal (Waldeyer's ring), and extra lymphatic extranodal (orbit and larynx) in terms of the affected tissues. The extralymphatic, extranodal manifestation is an uncommon finding in this region [1-4].

The differentiation of primary from secondary NHL of the larynx is essential for therapy decisions. Local radiotherapy is the mainstay for primary involvement, whereas secondary involvement indicates a higher stage and mandates systemic chemotherapy and immunotherapy [5, 6].

## Case presentation

A 72-year-old woman was referred to our department because of the sensation of a foreign body in her throat. She denied dysphagia, dyspnea, and B symptoms. She had a history of stage IVA MZL with evidence of IgG kappa monoclonal gammopathy, which was diagnosed 17 years ago. She received chlorambucil and prednisolone as therapy at the time. While receiving that therapy she developed a further orbital involvement of her lymphoma which was managed with local irradiation of the orbit with a total dose of 46.8 Gray. The patient obtained six courses of rituximab and bendamustin. Through the course of the therapy, she developed therapy-attributed myelodysplastic syndrome (t-MDS).

After the therapy, she expressed a stable disease status and was kept under the watchful waiting approach till the latest presentation. She also reported a history of idiopathic thrombocytopenic purpura (ITP) and arterial hypertension.

A fiberoptic laryngoscopy revealed a 2.5 x 2.5 cm round non-ulcerated mass arising from the left aryepiglottic fold in the supraglottic region. The mass was suspected to be a benign endolaryngeal polyp. No other pathological findings were detected. Due to technical issues on the day of the presentation, fiberoptic images could not be obtained during the laryngoscopy.

Laboratory blood tests revealed her lactate dehydrogenase value to be 397 U/L and pancytopenia.

**Figure 1:**
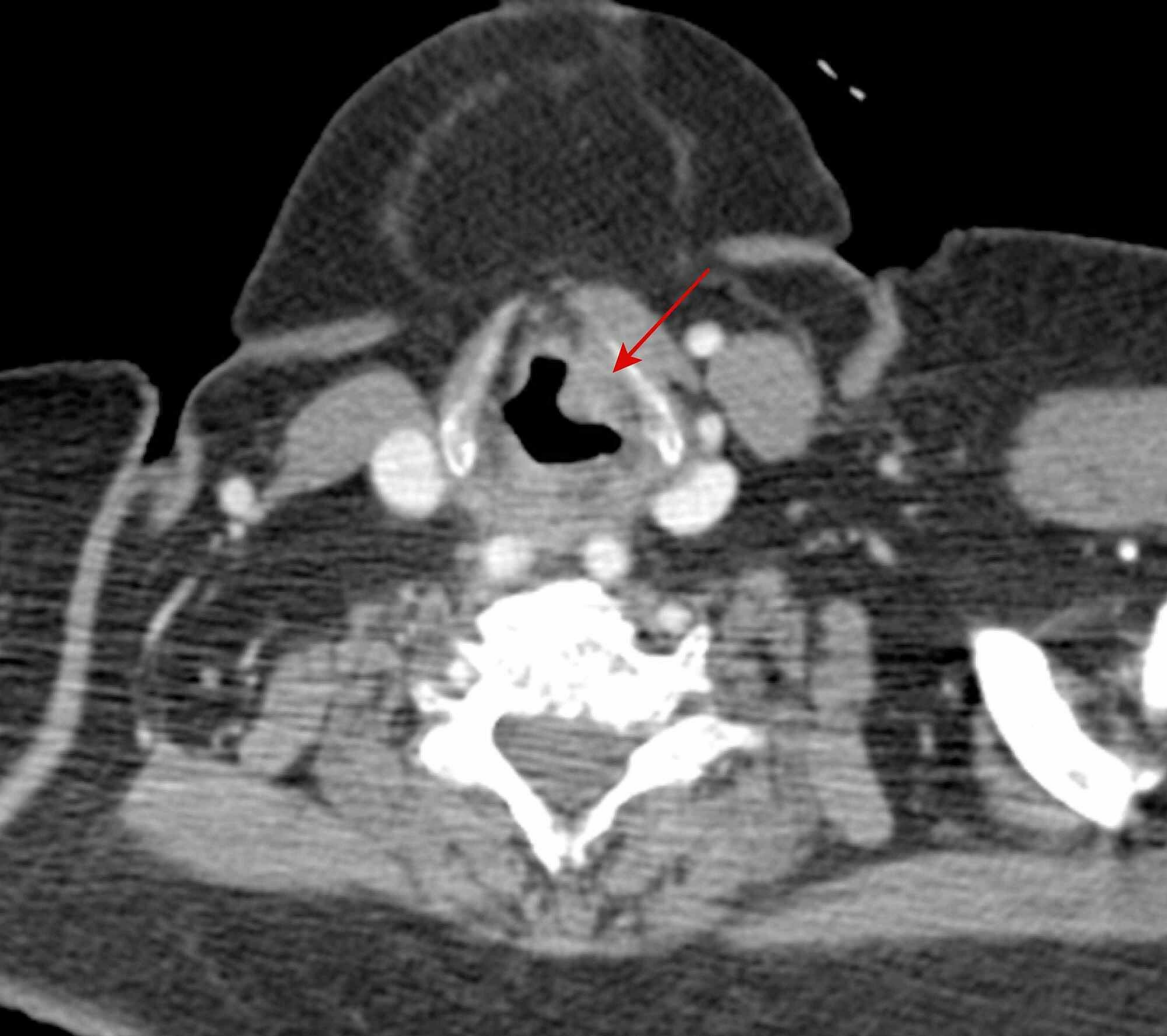
A CT scan with contrast (axial plane) conducted six months prior to surgery The mass is highlighted by the red arrow in the left supraglottic region.

Cancer staging with a full-body CT scan was performed six months prior to the presentation (Figure 1). During the scan an endolaryngeal mass was detectable. Unfortunately, the mass was not reported in the radiological report. The omission of the description of the mass in the radiological report may be attributed to the rarity of the finding and a lack of expertise in analyzing such an involvement of the larynx.

Since the endolaryngeal mass was suspected to be a polyp and the secondary MZL involvement of the larynx was deemed highly unlikely, an avoidable surgery was planned, and unfortunately, no PET-CT imaging was conducted.

The patient underwent microlaryngoscopic surgery under general anesthesia for resection and evaluation of the mass. Histopathological analyses were performed on the formalin-fixed, paraffin-embedded tissue sections of the resected mass. Lymphoid infiltration among normal epithelial cells was identified. Immunohistochemical staining showed a strong expression of CD20(+), Bcl6(-), CD10(-). Light chain Kappa restriction, about 20% CD138 (+) plasmacytoid cells, and proliferation fraction of about 20% were detected in the tumor cells. Molecular genetic analyses of the resected mass did not identify a mutation for Myd88 (exon 5). Based on the histopathological and molecular histological findings a diagnosis of marginal zone NHL was confirmed.

**Figure 2:**
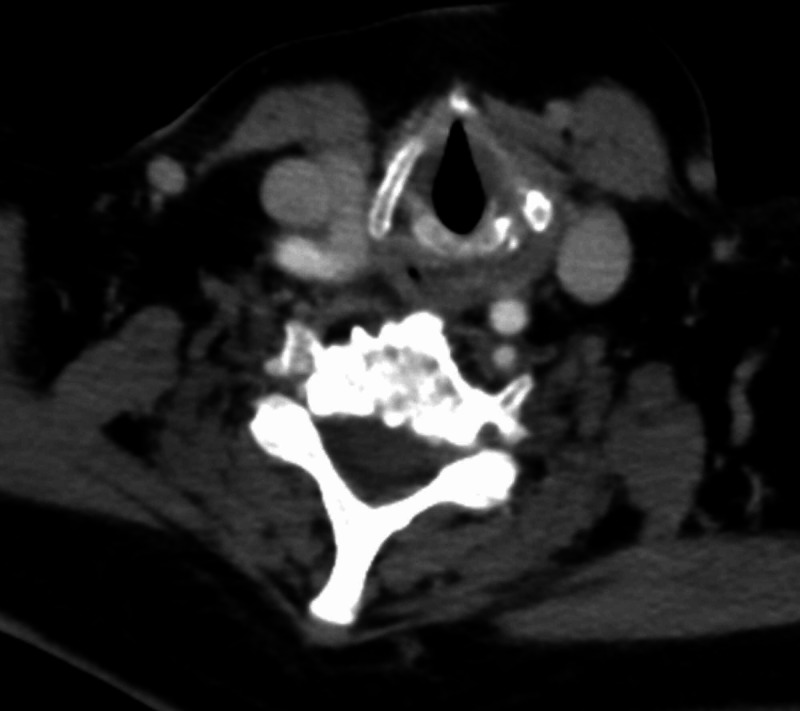
A CT scan with contrast (axial plane) conducted two weeks after the surgery No evidence of the mass is visible in the supraglottic region after the surgical resection.

For staging the patient underwent a bone marrow biopsy. The cytological and histological analyses showed no infiltration of the bone marrow with lymphoma but revealed a secondary acute myeloid leukemia (s-AML) phenotype M6. The s-AML developed secondary to the therapy (t-MDS). A contrast-enhanced full-body CT scan (compared to the CT scan performed six months earlier) revealed progressive lymph nodes in the posterior mediastinum which had been in partial remission previously. There was no evidence of the involvement of other lymph node groups. A previously known slight splenomegaly was stable. The CT scan shows no remnant of the initial endolaryngeal mass (Figure 2). Laboratory tests showed the persistence of the thrombocytopenia without evidence of platelet antibodies in immunological testing.

The patient was referred to the Department of Hematology and Oncology for continued management. Treatment was initiated with ibrutinib 520mg/d and rituximab 375 mg/m2 every 28 days.

Six and 12 months follow-ups with fiberoptic laryngoscopy showed no local recurrence of the endolaryngeal mass. A full-body CT scan showed a stable disease status.

## Discussion

The majority of secondary malignant involvements of the larynx arise from breast cancer or malignant lymphatic proliferation [7]. A secondary laryngeal manifestation of NHL is a rare finding as both primary and secondary involvement of the larynx account for less than 1% of all laryngeal neoplasms. Only about 100 cases of both findings have been reported in the literature [6, 7].

MZL is a less common type of B-NHL disease and contributes to 5-15% of all lymphomas in total. However, a retrospective study of 200 patients diagnosed with laryngeal lymphomas between 1973 and 2014 found interestingly that 16.5% of laryngeal lymphoma were MZL [4, 5].

The most common extranodal localizations of primary MZL are the stomach, followed by the adnexa and the orbit [3, 4]. Less than 15 cases of extranodal localizations of MZL were reported to involve the larynx according to a review published in 2009 [8].

The clinical features of our case are consistent with similar reported cases regarding presentation and clinical findings. There are no means to differentiate between primary and secondary involvement based only on clinical examination [6, 7]. In our case, we decided to resect the laryngeal mass suspecting a benign endolaryngeal lesion such as a polyp. We evaluated the secondary involvement of the larynx following MZL to be very unlikely. In hindsight, it seems that an MZL of the larynx may clinically mimic benign laryngeal lesions. If we had suspected the lesion to be secondary to MZL, we would have considered further specific imaging procedures like a PET-CT. A PET-CT would have detected the overall progress of the underlying MZL and spared the surgical excision.

While most of the published cases are about the primary involvement of the larynx with NHL, which indicates Stage IE according to the Ann Arbor staging system for lymphomas, our case describes a secondary involvement of the larynx with MZL which indicates Stage IV A [9]. Nonetheless, in our case, there was no upgrading of the staging because of the larynx involvement as the patient had already had stage IV A because of her previous orbital manifestation of MZL.

The differentiation between primary and secondary larynx involvement of MZL is paramount as it affects therapy regimes and prognosis [4]. Primary involvement of the larynx (Stage I E) MZL responds very well to local irradiation with or without chemotherapy [1, 4, 5, 8-10]. On the other hand, secondary involvement of the larynx represents a disseminated disease (Stage IV) which may mandate systematic chemotherapy in combination with immunotherapy [4, 6, 8]. Moreover, the higher stage associated with the secondary MZL involvement of the larynx indicates a poorer prognosis that can influence further management in patients with multimorbidities [4].

## Conclusions

In patients with MZL, it is paramount to thoroughly inspect uncommon sites such as the larynx for secondary involvement by radiological imaging. Misdiagnosing such an involvement can falsely downgrade the stage of the disease and delay proper treatment. Moreover, it may expose the patients to unnecessary surgical procedures. As benign endolaryngeal masses may mimic the MZL of the larynx clinically, a PET-CT scan before proceeding with the surgical planning is very helpful in differentiating MZL of the larynx from benign endolaryngeal lesions.

It is essential to differentiate primary from secondary involvement of the larynx in cases of malignant lymphatic proliferation as it has a great impact on further management, therapy, and prognosis. While local irradiation is the main therapy for primary involvement of the larynx, secondary involvement of the larynx in a disseminated disease may require a combination of systemic chemotherapy and immunotherapy.
